# Quantum Electrodynamics in High-Harmonic Generation: Multitrajectory Ehrenfest and Exact Quantum Analysis

**DOI:** 10.1021/acs.jctc.4c01206

**Published:** 2024-12-24

**Authors:** Sebastián de-la-Peña, Ofer Neufeld, Matan Even Tzur, Oren Cohen, Heiko Appel, Angel Rubio

**Affiliations:** † Max Planck Institute for the Structure and Dynamics of Matter, Luruper Ch 149, Hamburg 22761, Germany; ‡ Schulich Faculty of Chemistry, Technion - Israel Institute of Technology 3200003 Haifa, Israel; § Department of Physics and Solid State Institute, Technion-Israel Institute of Technology 3200003 Haifa, Israel; ∥ Center for Computational Quantum Physics, The Flatiron Institute, 162 fifth Ave, New York, New York 10010, United States

## Abstract

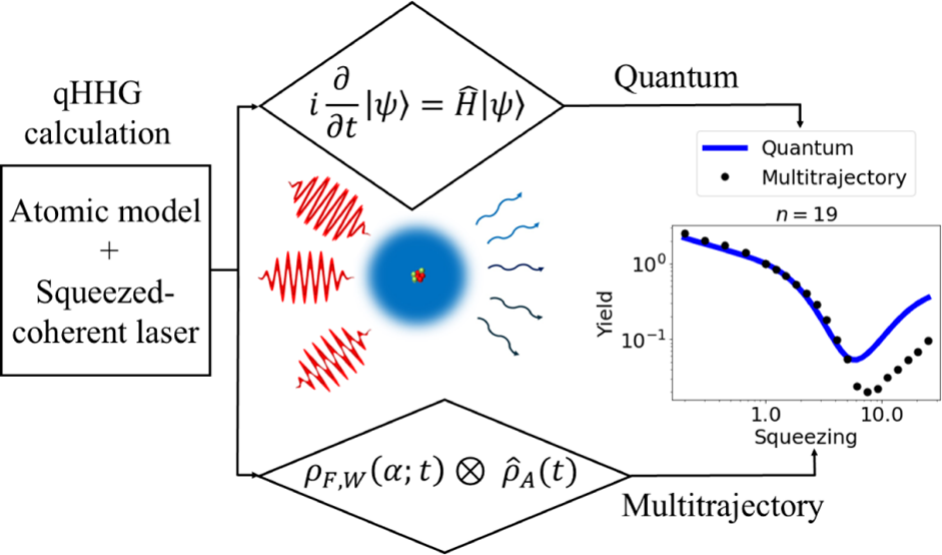

High-harmonic generation (HHG) is a nonlinear process in which a material sample is irradiated by intense laser pulses, causing the emission of high harmonics of incident light. HHG has historically been explained by theories employing a classical electromagnetic field, successfully capturing its spectral and temporal characteristics. However, recent research indicates that quantum-optical effects naturally exist or can be artificially induced in HHG, such as entanglement between emitted harmonics. Even though the fundamental equations of motion for quantum electrodynamics (QED) are well-known, a unifying framework for solving them to explore HHG is missing. So far, numerical solutions have employed a wide range of basis-sets, methods, and untested approximations. Based on methods originally developed for cavity polaritonics, here we formulate a numerically accurate QED model consisting of a single active electron and a single quantized photon mode. Our framework can, in principle, be extended to higher electronic dimensions and multiple photon modes to be employed in *ab initio* codes for realistic physical systems. We employ it as a model of an atom interacting with a photon mode and predict a characteristic minimum structure in the HHG yield vs phase-squeezing. We find that this phenomenon, which can be used for novel ultrafast quantum spectroscopies, is partially captured by a multitrajectory Ehrenfest dynamics approach, with the exact minima position sensitive to the level of theory. On the one hand, this motivates using multitrajectory approaches as an alternative for costly exact calculations. On the other hand, it suggests an inherent limitation of the multitrajectory formalism, indicating the presence of entanglement and true quantum effects (especially prominent for atomic and molecular resonances). Our work creates a roadmap for a universal formalism of QED-HHG that can be employed for benchmarking approximate theories, predicting novel phenomena for advancing quantum applications, and for the measurements of entanglement and entropy.

## 1 Introduction

High-harmonic generation (HHG) is a nonlinear optical process in which molecules,^1,2^ liquids,^3^ or solids^4^ are exposed to an intense light source and radiate higher harmonics of the driving light main frequency. This phenomenon has enabled the birth of new research areas like attosecond spectroscopy,^5,6^ and is routinely used for generating coherent X-rays table-top.^7^ Initially, HHG in atomic and molecular systems was understood as a consequence of the semiclassical motion of the electron around the nucleus^8^ and later it was explained using quantum mechanical models.^9−11^ In these models, the light source was treated as a classical electromagnetic field, making it inapplicable for analyzing HHG from emerging quantum light sources or for explaining quantum-optical effects and interferometry in the emitted harmonic spectra.

Recent research shows that quantum-optical effects are, in fact, potentially prominent in high-harmonic generation.^12,13^ This has motivated the development of new theories^14,15^ capable of accounting for nonclassical light sources^16−20^ or entanglement in the emitted harmonics, even when the source is treated classically.^21−25^ The field is also experiencing major experimental efforts,^12,13^ quantum optical signatures, high-order harmonics photon statistics, superbunching,^26^ e.g., applying quantum sources for HHG^27,28^ or exploring violations of Cauchy–Schwarz’s inequalities in the emitted light.^29^

The main equations describing such phenomena are known exactly from quantum electrodynamics (QED).^30,31^ Nonetheless, they cannot be practically solved without resorting to approximations of either the Hamiltonian or wave functions,^32^ which has already led to some developments in cavity materials engineering by making use of density functional theory.^33−35^ However, to date, multiple papers have predicted a variety of phenomena based on various methodologies and approximations, mostly ad hoc, and some not necessarily agreeing with each other. An *ab initio* solution of the HHG system is unfeasible even with only a single active electron due to the exponential scaling of the bosonic basis set for highly populated photon states. Such a large number of photon modes are essential in HHG that is driven by very intense lasers and causes the emission of a broad spectrum. Generally, one would want to exploit the success of semiclassical multitrajectory techniques in the field of quantum chemistry for the electron–phonon coupling,^36,37^ a formalism which has also been tested for electron-photon systems in the context of spontaneous emission,^38,39^ and employ such an approach for describing quantum HHG. The multitrajectory Ehrenfest dynamics (MTEF) approach should capture qualitative dynamics intuitively and presents a linear scaling with the system size, bridging the notions of classical electrodynamics with quantum optics and enabling its use for more complicated systems. Yet, it fails to provide an exact quantitative description of processes, e.g., wrong predictions of final state population in spontaneous emission processes^38,39^ or zero-point energy leakage^40^ (although some solutions for this latter problem have been suggested.^40^ In the context of HHG, no testing of MTEF or trajectory-based theories has previously been done, and that level of approximation is untested.

Here, we theoretically study HHG in a 1D atom model irradiated by an intense quantum light source. In order to introduce quantum-optical states of light for HHG, we couple the electron to the light field through two models: (i) an exact quantized single photon mode with the frequency of the HHG driving field, leading to a formally accurate quantum model of the dynamics; and (ii) MTEF that approximates the quantized photon mode via multiple semiclassical simulations sampling a quantum-optical distribution function. Both numerical methods can, in principle, be extended and employed in a universal theory for benchmarking approximations and making predictions, either by including many electrons into consideration in quantum chemistry codes or by adding multiple photon modes. We employ these methods in a 1D model atom and test the viability of MTEF by comparing HHG driven by squeezed-coherent light with different degrees of squeezing between both methods. We observe a characteristic minimum emerging in all harmonic orders vs the phase squeezing parameter, a phenomenon that MTEF only partially captures, potentially exposing true quantum effects in the light-matter entanglement that MTEF systematically neglects. Our work paves the path for the development of an *ab initio* framework for solving QED-HHG, and provides a quantitative prediction of the squeezing dependence of the HHG spectrum that can be used for novel quantum ultrafast spectroscopy and benchmark previous approaches. An experimental test of such a system could be proposed using a similar technique as in Ref. (27) but with squeezed-coherent irradiated light instead of bright-squeezed vacuum.

The manuscript is ordered as follows: in Section 2 we describe our theoretical approaches. The comparison between methods for quantum HHG is given in Section 3, as is a discussion of the results. Finally, Section 4 summarizes our results and presents a future outlook.

## 2 Computational Methodology

Let us begin by describing our main observable of interest, how it is extracted from calculations, and our motivation. The standard theory for HHG uses a coherent light pulse with field intensity α_0_: , where  is the displacement operator^41^ and  is the vacuum state of the driving photon mode. The main result of the standard HHG theory allows us to compute the electromagnetic emission from the Fourier transform of the electronic dipole .^11,14,42^ The dipole *d*(*t*) is governed by a time-dependent Schrödinger equation (TDSE) with a driving electric field , where ω_*L*_ is the laser frequency and the electric field operator  is in the interaction picture.^30,31^ In this theory, the mean field of the driving photon system completely determines the HHG spectrum and, consequently, nonclassical states of light like squeezed states  (where  is the squeezing operator^41^ and ξ is the degree of squeezing) would lead to the same spectrum owing to their irrelevance in the expectation value of the electric field . Any such potential numerical approach must include the contribution of the squeezing, ξ, into the dipole spectrum, *d*(ω), such that these effects are noticeable in the HHG energy spectrum, . We will now develop two methods that are in principle capable of describing quantum light effects in HHG, and compare their effects on the dipole spectrum *d*(ω): an exact single-mode quantum electrodynamical model (Section 2.1) and an approximate semiclassical multitrajectory model (Section 2.2). Notably, both models presuppose that photons live in a cavity, which enables the use of quantized photon modes and relies on methods developed for *ab initio* cavity electrodynamics.^19,30,31,33−35,38,43^

### 2.1 Single-Mode Quantum Electrodynamical Model

**Figure 1:**
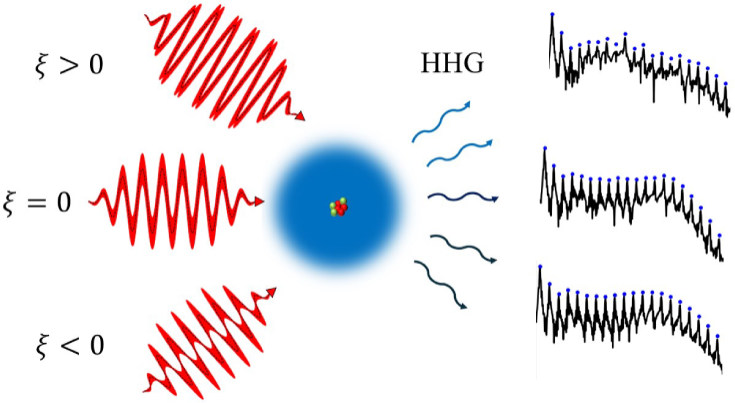
Schematic illustration of HHG driven by quantum light and leading to quantum optical effects. Laser fields with different squeezings (left) are irradiated onto an atom and this produces the HHG spectrum with potentially quantum features.

The Hamiltonian of our single quantized photon mode model consists of an electronic Hamiltonian , a single photon mode Hamiltonian , and the length-gauge dipole approximation interaction Hamiltonian (19,21) (we use atomic units unless stated otherwise):
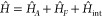
1where the electronic Hamiltonian is that of a one-dimensional model potential , with *b* the softening parameter and λ is the light-matter coupling (this coupling defines the cavity length  and physically describes the amplitude of vacuum fluctuations inside the cavity.^44^ This atomic model has been widely used in HHG works as it provides a qualitative description of gases, including comparison with the experimental results.^45−47^ The term  describes the self-interaction of the electronic dipole through the photon-mode in the length gauge.^30,31^ The free photon Hamiltonian  is that of a simple harmonic oscillator, where ω_*L*_ is the photon mode frequency. The choice of a single photon mode enables an exact solution that we can use to test the MTEF approximation. The operator  is related to the creation and annihilation operators of the photon mode via . Note that the free photon Hamiltonian can also be rewritten in terms of the creation and annihilation operators as . Finally, the length gauge interaction Hamiltonian is the dipole coupling to the electric field . The electric field operator of the photon is
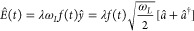
2such that the interaction Hamiltonian takes the form  if we write it in terms of the position operators  and . In this representation overall, the electronic coordinate is represented by the coordinate *x*, and the photonic coordinates by *y*. In eq 2, *f*(*t*) is the envelope of the light–matter interaction, which is off at the beginning of the simulation  and is smoothly turned on during the simulation time to , as is back off at the end of the simulation . The specific shape of *f*(*t*) (depicted in Figure 1 used in our simulation is
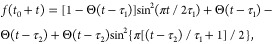
3where  is the Heaviside step-function,  and , thus the total simulation time is , which translates into 9 optical cycles with the full width at half-maximum (fwhm) being 8 optical cycles. It corresponds to a trapezoidal shape with smooth transitions. As an initial state, we choose the electronic ground state, , and a squeezed-coherent state for the photon mode, . The combined electron-photon initial state is then . This model allows fully entangled light-matter wave functions as the system evolves over time.

We employ Octopus code^48,49^ to solve the Schrödinger equation in this two-dimensional system (*x*, *y*) by expressing photon modes as 1D harmonics oscillators in the photon phase-space (eq 2). One advantage of this code is that it enables easy generalization to the many-electron case. For a multimode photon system, it would suffice to increase the dimensionality of the problem by adding more coordinates  to the Hamiltonian of eq 1. This has the disadvantage of exponential scaling but enables us to benefit from already implemented and optimized software that solves TDSE in N-dimensional systems, such as Octopus. At the very least, such an approach should be applicable for a single electronic coordinate, and up to five photonic ones, in accordance with efforts on exactly solving two-electron systems in 3D.^50^ While this is beyond our scope, adding photon modes could also be addressed using a different choice of basis sets, such as Fock or coherent states. Overall, this approach employs methods originally developed for quantum electrodynamics in cavities (e.g., for polaritonic chemistry,^51^ and repurposes them for quantum HHG by changing the boundary and initial conditions. This allows it to be implemented in typical quantum chemistry packages.

The numerical values of the parameters used in the simulation are the electron-photon coupling λ = 0.015 (corresponding to a photon cavity length of and a cavity fundamental frequency of  a.u.), the driving laser frequency  a.u. (corresponding to  and a wavelength of , widely used in HHG literature and experiments,^11,16,45,46^ the coherent intensity of the squeezed-coherent states  (which corresponds to a maximum electric field intensity of 0.053 a.u. or 10^14^ W/cm^2^,^11,16^ a squeezing parameter that is swept among a range of values , and the softening parameter for the electron model potential *b* = 0.816 a.u. (corresponding to a Neon ionization potential  a.u.). The converged parameters of the simulation are those of a 2D time-dependent simulation over time: electron box size  a.u., photon box size  a.u., electron finite-difference step d*x* = 0.7 a.u., photon finite-difference step d*y* = 0.1 a.u., time-step *dt* = 0.02 a.u., and complex absorbing potential with absorbing length for both coordinates *L*_*ab*_ = 30 a.u. (generally HHG is an open quantum system because electrons can photoionize and we can only describe a finite size system, so we add absorbing boundaries mimicking an open quantum system, which is a frequent technique in nonlinear quantum optics.^52^

Note that the numerical parameters are converged for this particular value of λ, as the light state is weighted by λ when analyzing the scale of the photon coordinate *y* (see eqs 2 and 9), which also affects convergence. Although this choice of λ is somewhat arbitrary, we will show below that the main minima feature in the HHG spectra exists for a wide range of values of λ, under the condition that a stronger coupling leads to a stronger effect of the squeezing in the HHG yield (see supporting information I). The particular value we used corresponds to medium light-matter coupling attainable within optical cavities,^38^ and is expected to be physical under reasonable experimental conditions (though the exact choice of λ heavily depends on the cavity geometry that the simulation employs).^19,24,38,44^ Importantly, λ is not a fully independent parameter in our simulation, as together with the choice of α_0_ it defines the expectation value of the laser peak power. Thus, whenever different values of λ are explored (see supporting information I), we also set α_0_ to keep the same peak field strength.

### 2.2 Multitrajectory Ehrenfest Description

Multitrajectory Ehrenfest dynamics (MTEF) is a model that approximates a quantum electrodynamical simulation with multiple semiclassical simulations that take into account the quantum uncertainty as classical statistical uncertainty,^38^ also analogous to including phonon modes.^37^ In our case, the photon mode is treated semiclassically while keeping the electronic system fully quantum, in-line with recent theories.^37−39^ MTEF approximates the combined electron-photon system to be unentangled (although some correlation is still captured), such that the combined density operator is , where  represents the density operator for the electron (atomic) system and  represents the density operator for the photon system. We expect improvements to the unentangled evolution between light and matter that MTEF presupposes, building upon recent work regarding the possibility of an exact-factorization procedure between light and matter.^53^ This contrasts with the single quantum photon mode of Section 2.1, which ensures that light-matter correlations are fully integrated.

We use the Wigner representation for the photon system,^41^ such that the photonic density operator becomes a function of the complex phase-space variable α (usually interpreted as the classical phase-space variable). The Wigner distribution of the photon mode is the analog of the density operator in the Wigner representation: , with . Expectation values are expressed as integrals over the phase-space variable α: , where  is the operator  in the Wigner representation.^41^ In the multitrajectory Ehrenfest algorithm, we approximate the Wigner distribution of the photon at the initial time  to be equivalent to a statistical sampling of *N*_traj_ trajectories α^*j*^, where *j* is the index of the trajectory, enabling a classical treatment of the photonic dynamics:
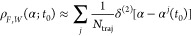
4where the complex Dirac delta can be understood as the product of the real and the imaginary Dirac delta: . The electron-photon system density operator  (whose photon part is expressed in the Wigner representation) takes the form:^38,39^
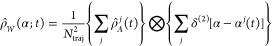
5where  are the electronic wave functions that evolve dynamically with the classical photon mode  for each respective trajectory *j*. The dynamical evolution of the coupled electron and photon system is given by the semiclassical set of equations (in contrast to the fully quantum Hamiltonian of eq 1):^30,31,38,39^
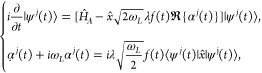
6where  is the Hamiltonian for the electronic system, which is the same as in eq 1. The initial conditions for each photon trajectory  are sampled from the Wigner distribution of the initial photon state . We will assume that there is no feedback from the electronic system into the classical photon trajectories, that is,  in eq 6. The evolution of the photon modes becomes that of free Maxwell equations: . By substituting this into eq 6, we finally reach the TDSE for the electronic system coupled to the classical photon mode:
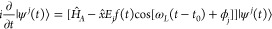
7where  and  are the field amplitude and phase, respectively, for the corresponding trajectory *j*, with  the electric field for this trajectory. The expectation values of the electron operators can be computed via the average response from the individual and independent trajectories (eq 5):
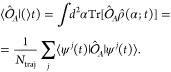
8

For squeezed-coherent states, the Wigner distribution has an analytical expression which corresponds to a Gaussian distribution  defined by the parameters of its coherence and squeezing, α_0_ and ξ, respectively:^16,41^
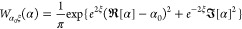
9

The squeezing parameters ξ and will be used interchangeably throughout the manuscript. Three cases of ξ are relevant for this work: ξ < 0 or s < 1 correspond to amplitude-squeezing, ξ = 0 or *s* = 1 correspond to no-squeezing (coherent state), and ξ > 0 or *s* > 1 correspond to phase-squeezing. Since we will use squeezed-coherent states as the initial photon states in our simulation, , the initial Wigner distribution of our photon modes is defined by eq 9: , which will be used for the sampling of the initial values of the photon phase-space variable .

The single quantized photon mode runs in approximately 20 min on one CPU, while the multitrajectory simulations require 30 s per trajectory on one CPU, and the MTEF simulation reaches convergence at around 10000 trajectories. The relative efficiency of MTEF compared to full QED simulation relies on the easy possibility to avoid the expontential scaling of the bosonic basis sets (already for two photon modes, we expect MTEF to be substantially faster and less computationally heavy than solving the quantum dynamics exactly).

## 3 Computational Results and Discussion

The observable to be compared between the two simulations is the dipole moment of the electron *d*(*t*) for different squeezings ξ, and the resulting HHG spectra, computed as an expectation value of the electron-photon time-dependent wave function for the QED model: , where ; and using a sum of expectation values for MTEF:  (eq 8), where the phase-space variable for each trajectory,  (see eq 7), has been sampled from a Wigner distribution of its corresponding squeezing  (eq 9) for MTEF. The dipole variable  is then Fourier transformed into the harmonic spectrum  also masked by the envelope of the incident field:^21^
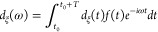
10

**Figure 2:**
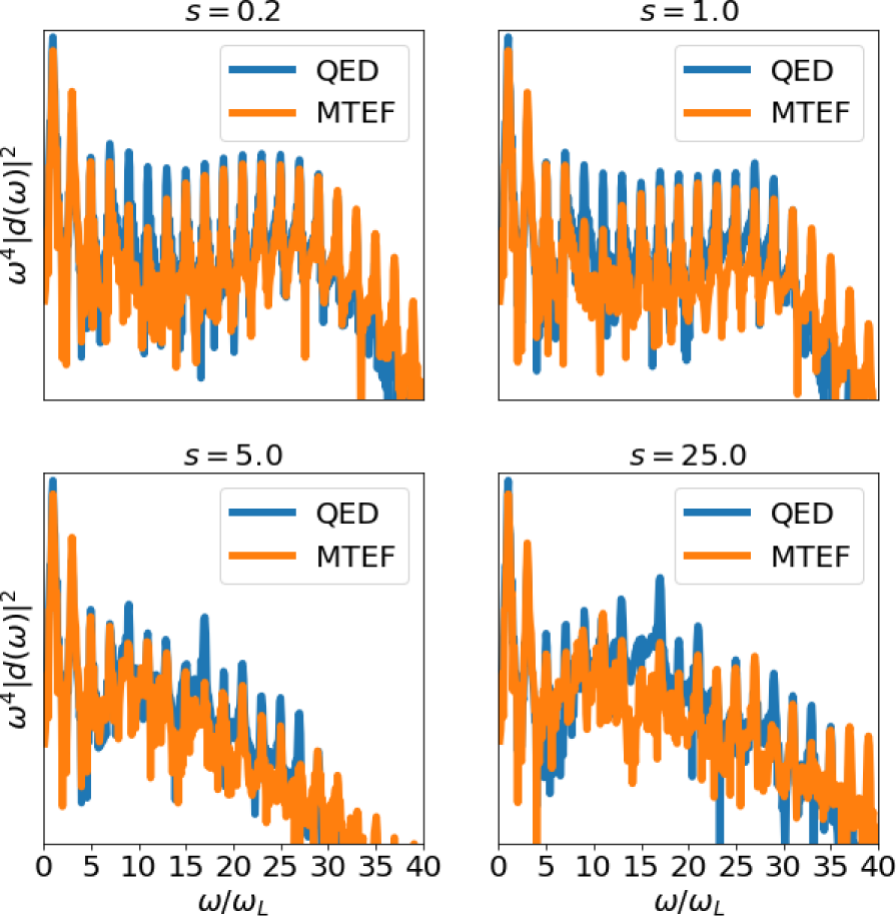
HHG emission spectra (in semilogarithmic scale) for different values of the squeezing parameter , 1.0, 5.0, and 25.0; and comparing both methods QED (blue) and MTEF (orange). As we enter into the phase-squeezing regime *s* > 1 a loss of the typical plateau () is observed, predicted by both methods (QED and MTEF).

Figure 2 presents the calculated HHG emission in semilogarithmic scale vs the harmonics  from both methods in typical conditions, and for different values of the squeezing parameter , 1.0, 5.0, and 25.0. The data in Figure 2 predict that phase-squeezing removes the plateau (11 < *n* < 31) and, instead, manifests a consistent drop in its HHG yield (a phenomenon observed already in previous semiclassical works.^18^Figure 2 reveals some discrepancy between MTEF and QED, which suggests true quantum effects beyond semiclassical interpretations play a role in the dynamics and HHG emission mechanism.

**Figure 3:**
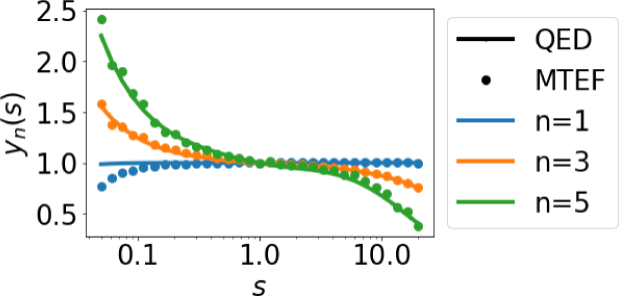
Normalized yield for the first [], third [], and fifth harmonics [] for the quantum electrodynamical simulation (QED, solid lines) and the multitrajectory Ehrenfest dynamics (MTEF, circles) vs the squeezing parameter .

**Figure 4:**
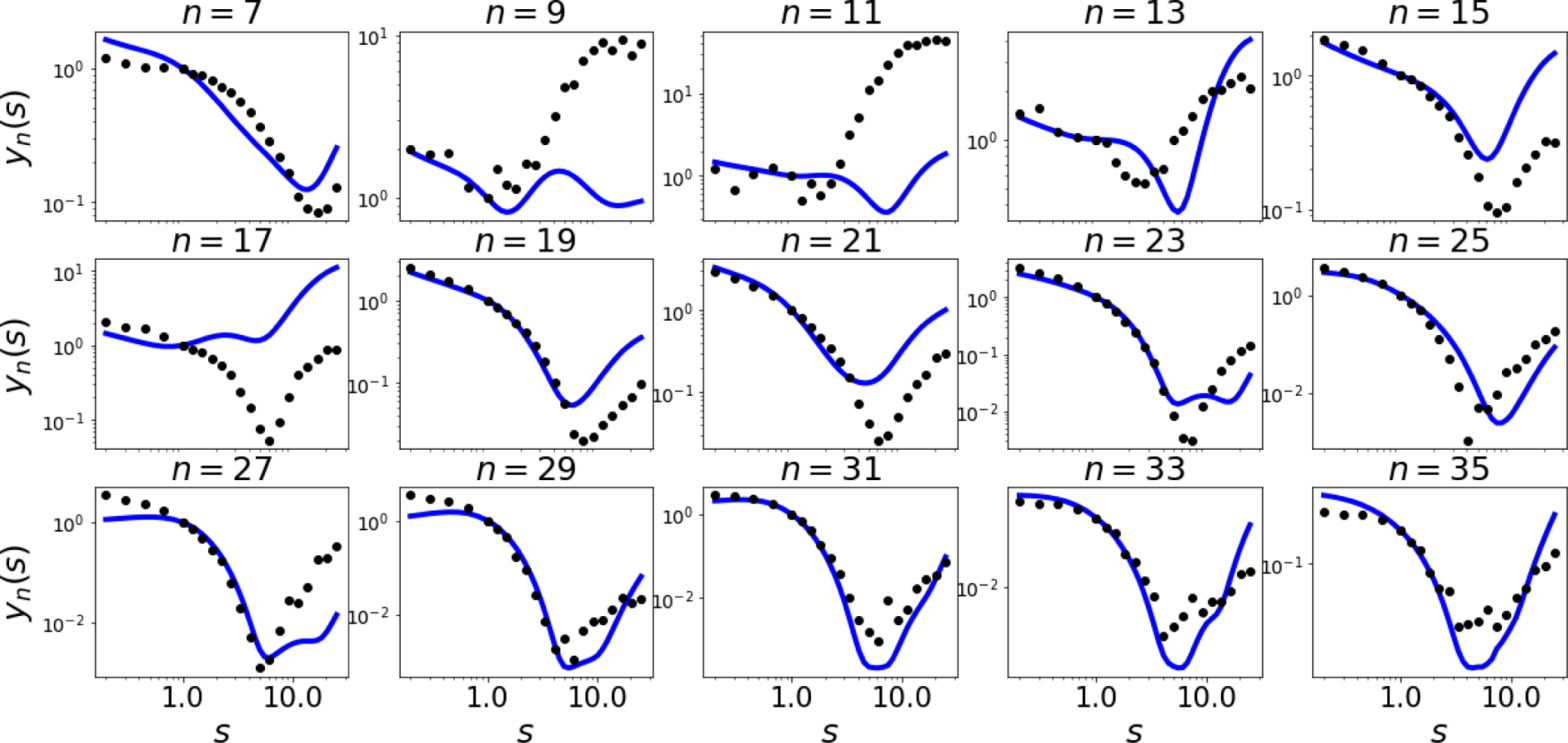
Normalized HHG yield for the quantum electrodynamical simulation (QED, solid blue line) and the multitrajectory Ehrenfest dynamics (MTEF, black circles) from the seventh [] to the 35th harmonic [] vs the squeezing parameter defined as . In all of the harmonics, we observe a minimum at a phase-squeezing value *s* > 1 (ξ > 0), which is captured by both methods.

The spatial symmetry of the electronic model potential together with that of the incident light forbids even harmonics of ω_*L*_ from being emitted^9,14,54^ (to observe phenomena like sum frequency generation, a molecular system that breaks spatial symmetry is required). Consequently, for analyzing the HHG yield, we integrate the dipole spectrum from even harmonic to even harmonic to get the harmonic-order-resolved yield  (Figure 2). We compare the normalized yield  for different squeezings, that is, the yield of the dipole emission for different harmonics  normalized to the dipole emission of the coherent state : . The computational results comparing the quantum electrodynamical simulation and the multitrajectory Ehrenfest dynamics are shown in Figures 3 and 4 for each of the harmonics from the first to the 35th (the cutoff is at around ), where we see the normalized yield  vs squeezing .  is particularly hard to test for MTEF (as opposed to simpler observables such as forbidden harmonics, cutoff scalings, etc.), but it is an interesting variable to analyze the effects of squeezing in HHG.

We observe from Figure 3 that the changes in the low-order perturbative harmonics (those before the HHG plateau) due to squeezing (solid line) are fully captured by the statistical distribution of the initial squeezed-coherent state (round dots), which means that the effects of the squeezing in these harmonics can be explained using the initial Wigner distribution of eq 9 through MTEF. This suggests that lower harmonics can be described by unentangled light-matter dynamics, although the first harmonic shows some deviation (a phenomenon that we currently do not understand). Figure 3 HHG predicts, in addition, that there is an increase in the yield for amplitude-squeezing and a decrease in yield for phase-squeezing.

**Figure 5:**
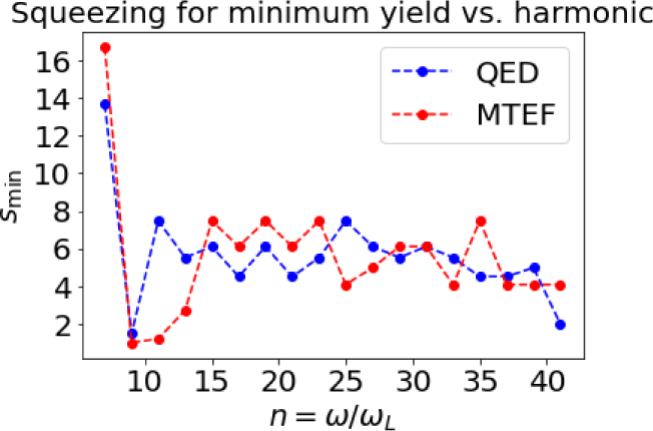
Squeezing  at which the yield is minimum vs harmonic order for both simulations QED (blue) and MTEF (red).

Figure 4 presents the main physical results of this paper analyzing the HHG yield for higher order harmonics vs squeezing: (i) We numerically observe a clear spectral minima behavior vs squeezing that is universal for all harmonic orders (see Figure 5). This feature and the exact minima position is expected to be highly sensitive to the system parameters (e.g., laser regime and electronic structure), which makes it potentially useful for developing novel ultrafast quantum spectroscopies. Notably, the existence of the minima themselves seems to be robust with different system parameter regimes. (ii) The MTEF simulation only partially reconstructs this minima structure (e.g., in harmonics 19–35, but failing in harmonics 11 and 17, and often missing the exact squeezing value for which the minima is obtained). Remarkably, this disagreement between MTEF and QED means that the approximations in MTEF are likely too strong for HHG driven by squeezed light, and hints that true entanglement plays a role in HHG emission.^55^ Let us further emphasize that the minima structure is also attained for almost all harmonics in a wider range of coupling strength (see supporting information I), but the position of the minima differs between the chosen parameters.

In further analysis, we find that the squeezing value at which the HHG minima is found is numerically close to the minima of the instantaneous correlation functions of the photon initial states  for , which might be explained by the multiphoton processes that are involved in the dipolar emission of these harmonics.^28^ However, we found that this value does not match the scaling of the minima position with the laser parameters such as intensity and wavelength (see supporting information II), meaning it likely does not capture the physical mechanism causing it.

Two harmonics are especially important for the analysis: *n* = 9 and *n* = 11. These harmonics are resonant with the electronic system (close to a transition in the electronic system), and this might explain the much more pronounced difference between MTEF and exact quantum dynamics for these energies as this resonance could be the source of high light-matter correlation, not covered in the MTEF model.^25^ Thus, our numerical results, which show a particularly strong disagreement between MTEF and full quantum simulations for resonance harmonics, suggest the potential for strong quantum optical effects in molecular or atomic resonances.^56,60^ Our results reveal the capacity of MTEF to predict many of the qualitative effects that squeezed light causes on the electronic dipole, but they simultaneously also expose the limitation of the current description of MTEF to cover all the quantitative effects observed in full QED simulations.

## 4 Conclusion

We explored quantum HHG simulations comparing two different methods for ultrafast electronic dynamics under a strong and squeezed driving field: an exact quantum electrodynamical model using a single quantized photon mode for the driving field and an approximate semiclassical multitrajectory Ehrenfest simulation. We tested the MTEF semiclassical approximation for quantum HHG, concluding that it partially captures the squeezing-dependence of the HHG yield. MTEF is able to explain many of the changes that occur in the HHG yield due to the squeezing, e.g., the existence of a characteristic minima structure, and especially the behavior of perturbative harmonics. This result provides a milestone on how HHG with quantum light could be tested in future systems and paves the way to a universal framework of QED-HHG. Moreover, our result reveals that phase-squeezing qualitatively affects the HHG spectrum by removing the characteristic plateau structure, manifesting instead an irregular pattern of decreasing HHG yield, a phenomenon that both methods (QED and MTEF) predict. However, we additionally find that not all of the HHG spectral features can be explained by our MTEF simulations, which hints that true quantum effects are required for explaining the full electron-photon interaction. In particular, the exact position of HHG minima vs squeezing is sensitive to the level of theory, suggesting it could provide an emerging observable in novel ultrafast quantum spectroscopies, as well as to benchmark new theories and approximations (especially near resonances that could serve as novel platforms for entanglement^56−60^ Looking forward, our work should motivate further theoretical developments and proposes an experimental setup and test to benchmark theory and uncover quantum effects in HHG.

## Supporting Information Available

I: light-matter coupling analysis; II: testing the correlation function hypothesis (PDF)
