# Supplementary material for: Quantum Electrodynamics in High-Harmonic Generation: Multitrajectory Ehrenfest and Exact Quantum Analysis
Source: J Chem Theory Comput. 2024 Dec 24;21(1):283–90. doi: 10.1021/acs.jctc.4c01206 (PMC11736686; doi:10.1021/acs.jctc.4c01206)
Supplement: Supplementary file 1 — ct4c01206_si_001.pdf [file ct4c01206_si_001.pdf]

Supplementary information for quantum electrodynamics in high harmonic generation: multi-trajectory Ehrenfest and exact quantum analysis

Sebastian de-la-Peña[1] *, Ofer Neufeld[1,2], Matan Even Tzur[3], Oren Cohen[3], Heiko Appel[1], and Angel Rubio[1,4]
[1] *Max Planck Institute for the Structure and Dynamics of Matter, Luruper Ch 149, 22761 Hamburg, Germany*
[2] *Schulich Faculty of Chemistry, Technion - Israel Institute of Technology, , 3200003 Haifa, Israel*
[3] *Department of Physics and Solid State Institute,*
Technion–Israel Institute of Technology, 3200003 Haifa, Israel and
[4] *Center for Computational Quantum Physics, The Flatiron Institute, 162 5th Ave, New York, NY 10010, USA*
(Dated: December 5, 2024)

* `sebastian.delapena@mpsd.mpg.de`

I. Light-matter coupling analysis

The light-matter coupling λ of Eq. (2) and (6) is a parameter that we presuppose in our model, as we are using a single photon mode in a cavity. We use the value $\lambda = 0.015$ in accordance with ref. [38]. This section is meant to provide a brief discussion on the consequences of changing the value of λ. For our 1D cavity modes, the value of the light-matter coupling is related to the size of the cavity L_c through: $\lambda = \sqrt{8\pi/L_C}$ [38, 39] (analogous to the quantization volume in 3D models [19, 24, 30, 31])

Figure S1 presents exemplary HHG spectra for various values of λ (changing by $\pm 33\%$ for the value in the main text). The HHG spectra is largely unaffected by these changes, supporting the generality of our conclusions, and indicating our predictions would hold in a wide range of experimental conditions. At very high squeezing values (Fig. S1 for $s = 25.0$), stronger changes begin to emerge in the harmonic spectra, especially beyong the cutoff. This agrees with the semiclassical interpretation of the photon mode Wigner distribution [Eq. (9)], since a higher λ means that the effects of the squeezing on the matter system are amplified, and thus for a given value of phase-squeezing we obtain higher electric field fluctuations (they scale as $\Delta E_{\max} \sim \lambda e^{\xi}$) in relation to the laser amplitude that remains constant at $E_0 = 0.053$ a.u.. We observe, nontheless, that the HHG minimum vs. squeezing is still present for these values of the light-matter coupling for almost all harmonic orders. In real measurements, the value of λ should be determined experimentally.

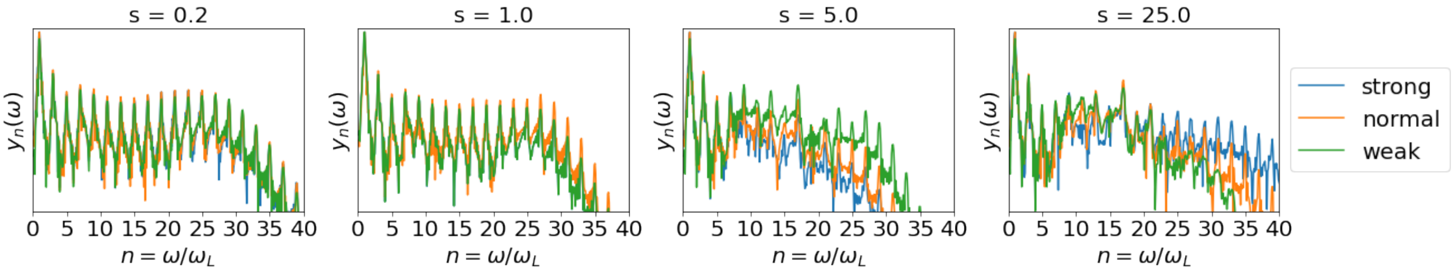

FIG. S1. HHG yield using three values of the light-matter coupling λ: strong coupling $\lambda = 0.020$, medium coupling $\lambda = 0.015$ and weak coupling $\lambda = 0.010$. Four values of the squeezing are given $s = e^{2\xi} = 0.2$, 1.0, 5.0, 25.0.

II. Testing the correlation function hypothesis

The minima structure observed for most of the harmonics in Fig. 4 motivated an analysis of what exactly was causing this phenomenon. It turns out that this minima structure is observed also in the nth-order instantaneous correlation function of the initial squeezed-coherent state $g^{(n)}(\xi)$ vs. the squeezing of this state for a fixed α_0 [42]:

$$g^{(n)}(s) = \frac{\langle \alpha_0, \xi | \, \hat{a}^{\dagger n} \hat{a}^n \, | \alpha_0, \xi \rangle}{\langle \alpha_0, \xi | \, \hat{a}^{\dagger} \hat{a} \, | \alpha_0, \xi \rangle^n},$$

where $s = e^{2\xi}$. If we consider, for the sake of testing this hypothesis, running the single quantum photon mode simulation for various laser intensities (correspoding to varing α_0) we can obtain the resulting minima in the HHG yield as we did in Fig. 4 (which would be the case $\alpha_0 = 0.053$). We now compare the phase-squeezing value $s_{\min}$ at which this is observed for various α_0 with the minimum of the correlation function for the corresponding initial state. The results of this comparison are shown in Fig. S2, where we observe that the trend of the HHG yield is not completely described by the correlation function, even though the numerical values and the increasing tendency vs. electric field intensity do have some qualitative agreement. This suggests that this hypothesis could still be ruled out in future research as a true mechanism to explain the results in HHG yield, even though it does reveal a numerical correlation for the parameters of our simulation, which has already been verified for lower harmonics in [28].

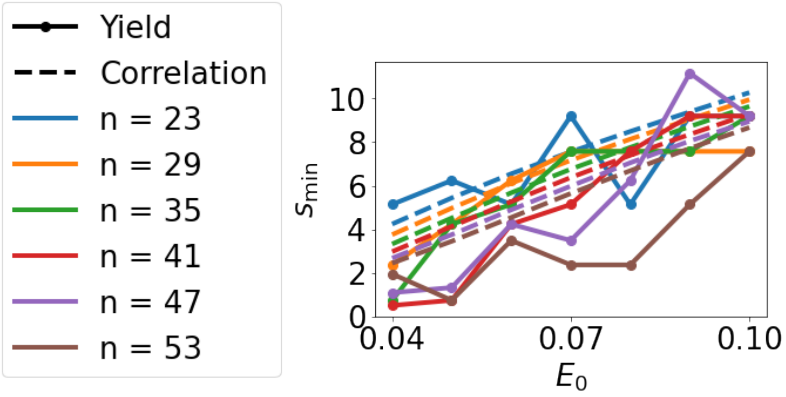

FIG. S2. Comparison of the phase-squeezing minima $s_{\min}$ vs. the power of the laser $E_0 = \sqrt{2\omega_L}\lambda\alpha_0$, for the single mode QED numerical simulation (dots) and the correlation function $g^{(n)}(s)$ [dashed line]. The number n represents the yield harmonics we are considering and the order of the correlation function, respectively.
